# Genome response to tissue plasminogen activator in experimental ischemic stroke

**DOI:** 10.1186/1471-2164-11-254

**Published:** 2010-04-21

**Authors:** Glen C Jickling, Xinhua Zhan, Bradley P Ander, Renée J Turner, Boryana Stamova, Huichun Xu, Yingfang Tian, Dazhi Liu, Ryan R Davis, Paul A Lapchak, Frank R Sharp

**Affiliations:** 1 Department of Neurology and M.I.N.D. Institute, University of California at Davis, Sacramento, California 95817, USA; 2 Department of Neurology, Cedars-Sinai Medical Center, Los Angeles, California, 90048, USA

## Abstract

**Background:**

Tissue plasminogen activator (tPA) is known to have functions beyond fibrinolysis in acute ischemic stroke, such as blood brain barrier disruption. To further delineate tPA functions in the blood, we examined the gene expression profiles induced by tPA in a rat model of ischemic stroke.

**Results:**

tPA differentially expressed 929 genes in the blood of rats (p ≤ 0.05, fold change ≥ |1.2|). Genes identified had functions related to modulation of immune cells. tPA gene expression was found to be dependent on the reperfusion status of cerebral vasculature. The majority of genes regulated by tPA were different from genes regulated by ischemic stroke.

**Conclusions:**

tPA modulates gene expression in the blood of rats involving immune cells in a manner that is dependent on the status of vascular reperfusion. These non-fibrinolytic activities of tPA in the blood serve to better understand tPA-related complications.

## Background

Tissue plasminogen activator (tPA) is currently the only approved medical therapy for acute ischemic stroke. Its use is limited to the first 3-4.5 hours after stroke onset due to an increased risk of complications beyond this time window such symptomatic hemorrhagic transformation (HT) [1]. Factors predisposing to tPA related HT remain to be fully defined, though several features are associated with increase HT risk including time from stroke onset, hypo-attenuation on pretreatment CT, leukoaraiosis, NIHSS on admission, diabetes, high systolic blood pressure, age, and thrombocytopenia [2,3]. tPA acts by cleaving plasminogen into the active enzyme plasmin, which degrades fibrin-based clots. The half-life of tPA's proteolytic activity on plasminogen is 3-7 minutes. In addition to tPA's fibrinolytic activity, a number of non-fibrinolytic properties have been described for tPA, some of which predispose to HT, blood brain barrier (BBB) disruption, and neuronal injury [4-7]. In this study, we sought to better define tPA's effects in acute ischemic stroke, determine if there is a blood gene expression profile that correlates with tPA administration, and determine if this blood gene expression profile differs from the profile associated with ischemic stroke.

Gene expression profiles are a useful tool to study complex diseases, including ischemic stroke, as shown in previous studies of blood gene expression in humans and animals [8-10]. The rationale for gene expression changes in the blood following ischemic stroke is largely based on the immune response to ischemic brain that is distinct from other neurological conditions [11,12]. Gene expression is also affected by medications, as observed with aspirin in ischemic stroke and corticosteroids in Duchenne muscular dystrophy [8,13].

Using a rat model of ischemic stroke, we performed gene expression analysis on whole blood samples to determine the expression profile for tPA. A difference in gene expression was observed between rats receiving tPA compared to rats not receiving tPA. Some genes expressed following administration of tPA interacted with ischemic stroke, with a different gene expression profile for tPA in rats with middle cerebral artery occlusion (MCAO) followed by reperfusion compared to rats with permanent-MCAO. Despite the interaction between tPA and ischemic stroke, the majority of the genes regulated by tPA can be distinguished from genes regulated by ischemic stroke.

## Results

There were no significant differences in physiologic parameters between animals receiving tPA compared to those not receiving tPA (data not shown). The median neurological deficit score was 3 for both reperfused-MCAO groups, with no significant difference between rats treated with or without tPA (p = 0.32). The median neurological deficit score was 3 for both permanent-MCAO groups, with no significant different between rats treated with or without tPA (p = 0.32).

### Analysis 1. tPA and Ischemic stroke

Gene expression associated with tPA administration was examined in an ANOVA model including tPA, ischemic stroke, anesthesia, and the interaction between tPA and ischemic stroke. tPA, ischemic stroke, and the interaction between tPA and ischemic stroke were found to affect gene expression.

#### tPA gene expression

There were 929 gene probes differentially regulated by tPA (p ≤ 0.05, fold change ≥ |1.2|) (Additional File 1). Of these 929 gene probes, 391 were up regulated (42.1%) and 538 were down regulated (57.9%). Functional analysis was performed using IPA. The 929 gene probes mapped to 660 annotated genes and generated a total of 514 network eligible genes. Among the top functions were cell signaling, cellular assembly and organization, inflammatory response, and cell death. Genes of interest include MMP9 (-1.84 fold), TLR2 (-1.38 fold), IL1R2 (-1.63 fold) and CAMK4 (+1.35 fold) (Table 1-gene symbol abbreviations).

**Table 1:** List of gene symbol abbreviations.

**Gene Symbol**	**Entrez Gene Name**		**Gene Symbol**	**Entrez Gene Name**
		
ANXA1	annexin A1		ITGB2	integrin, beta 2 (complement component 3 receptor 3 and 4 subunit)
		
ANXA2	annexin A2		JAG1	jagged 1 (Alagille syndrome)
		
BCL6	B-cell CLL/lymphoma 6		LY96	lymphocyte antigen 96
		
CAMK4	calcium/calmodulin-dependent protein kinase IV		ORAI1	ORAI calcium release-activated calcium modulator 1
		
COL17A1	collagen, type XVII, alpha 1		PIK3CD	phosphoinositide-3-kinase, catalytic, delta polypeptide
		
CSF1	colony stimulating factor 1 (macrophage)		TIMP3	TIMP metallopeptidase inhibitor 3
		
F5	coagulation factor V (proaccelerin, labile factor)		TLR2	toll-like receptor 2
		
FCGR2B	Fc fragment of IgG, low affinity IIb, receptor (CD32)		XDH	xanthine dehydrogenase
		
FGR	Gardner-Rasheed feline sarcoma viral (v-fgr) oncogene homolog		MMP9	matrix metallopeptidase 9 (gelatinase B, 92kDa gelatinase, 92kDa type IV collagenase)
		
GZMB	granzyme B (granzyme 2, cytotoxic T-lymphocyte-associated serine esterase 1)		SELL	selectin L
		
ICAM2	intercellular adhesion molecule 2		SERPINB6	serpin peptidase inhibitor, clade B (ovalbumin), member 6
		
IL1R2	interleukin 1 receptor, type II		SLPI	secretory leukocyte peptidase inhibitor

#### tPA and Ischemic Stroke

**Figure 1:**
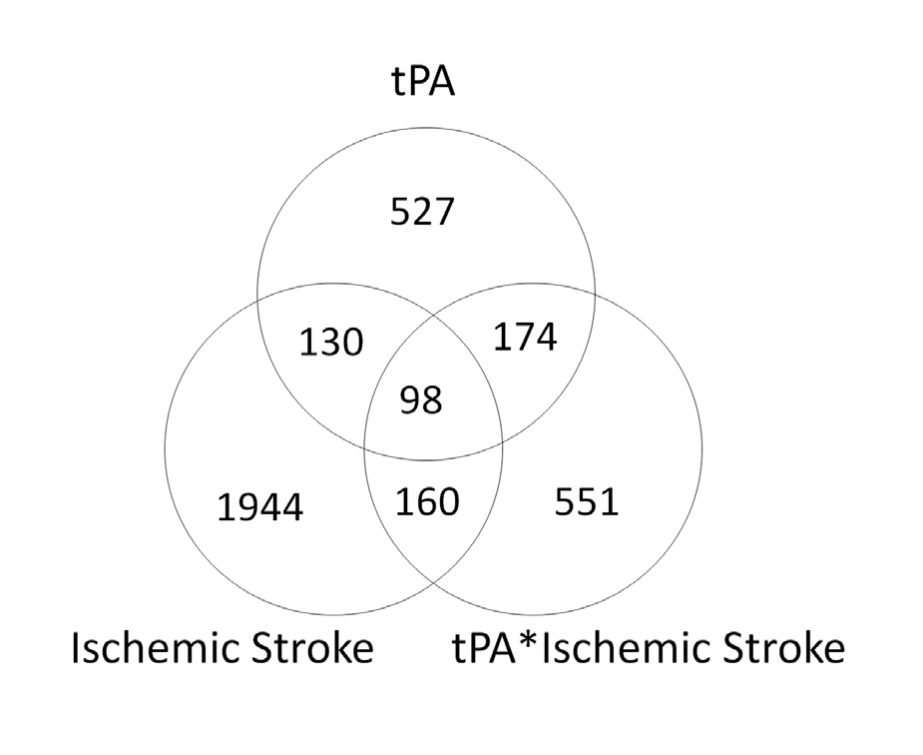
**Venn diagram of numbers of gene probes differentially regulated by tPA, ischemic stroke, and tPA-ischemic stroke interaction from the ANOVA analysis (p ≤ 0.05, fold change ≥ |1.2|)**.

An interaction was identified between tPA and ischemic stroke. There were 2332 gene probes regulated by ischemic stroke and 983 gene probes regulated by the interaction of ischemic stroke with tPA (p ≤ 0.05, fold change ≥ |1.2|) (Figure 1). There were 130 genes regulated by ischemic stroke and tPA, 527 genes regulated by tPA and not by ischemia, and 1944 genes regulated by ischemic stroke that were not regulated by tPA (p ≤ 0.05, fold change ≥ |1.2|).

### Analysis 2. tPA gene expression depends on the status of vascular reperfusion

#### tPA gene expression in rats with reperfused-MCAO

**Figure 2:**
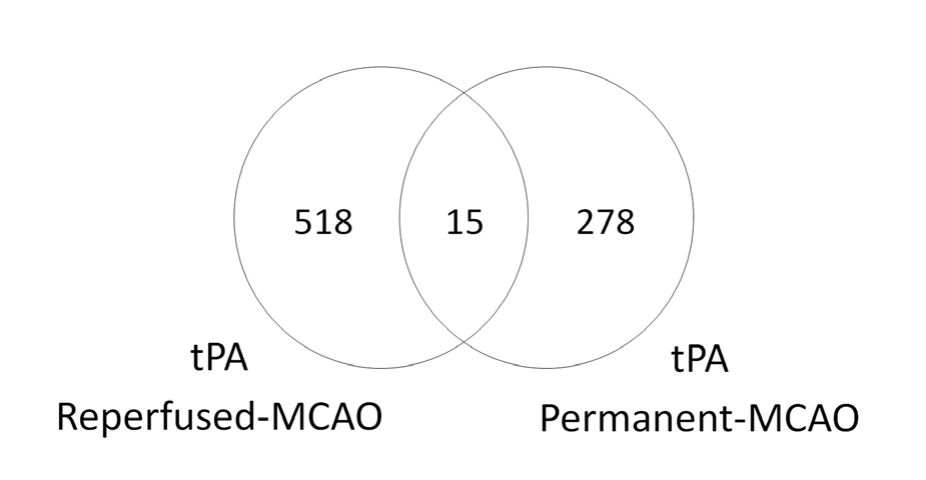
**Venn diagram of gene probes differentially regulated by tPA in rats with a 2 hour MCAO followed by 22 hours of reperfusion (Reperfused-MCAO) and rats with permanent 24 hour MCAO without reperfusion (Permanent-MCAO) (p < 0.01, fold change of ≥ |1.2|**. (MCAO = middle cerebral artery occlusion)

**Figure 3:**
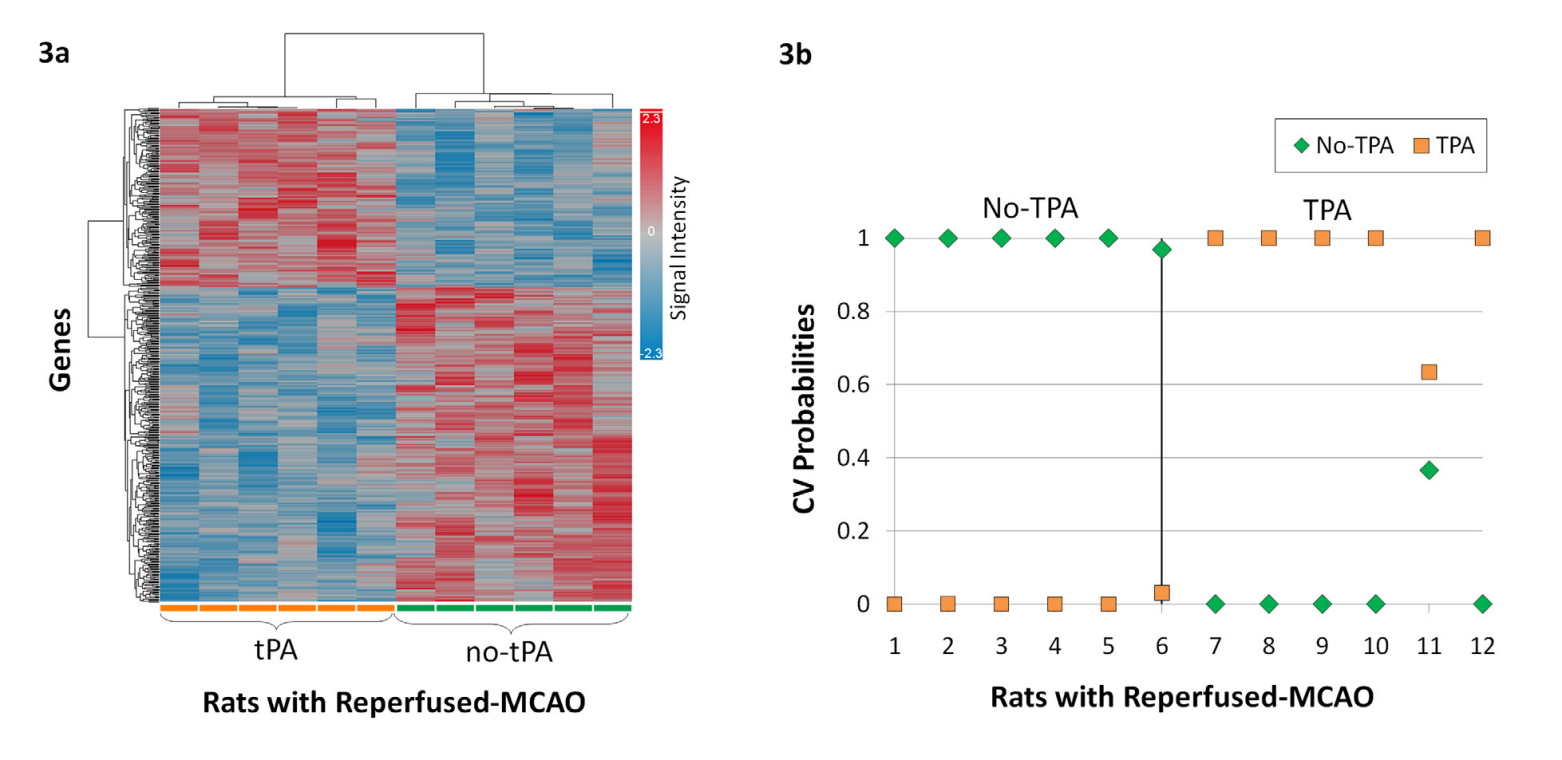
**Analysis of gene probes differentially regulated in the blood of rats with reperfused-MCAO (p ≤ 0.01, fold change ≥ |1.2|)**. (3a) Hierarchical cluster plot showing gene probes clustering by treatment with tPA. (3b) Cross-validation with Prediction Analysis of Microarrays (PAM) of gene probes differentially regulated by tPA. The 27 gene probes correctly predict treatment with tPA. (Rats not treated with tPA are shown on the x-axis by numbers 1-6, rats treated with tPA are number 7-12; y-axis represents the cross-validated probability of the predicted class)

In rats with reperfused-MCAO, 533 genes probes were differentially regulated by tPA at 24 hours (p ≤ 0.01, fold change ≥ |1.2|) (Additional File 2). The 533 gene probes comprised 193 up regulated (36.2%) and 340 down regulated (63.9%) gene probes. Of these 533 gene probes, only 15 overlapped with gene probes expressed by tPA in rats with permanent-MCAO (Figure 2). The 533 gene probes clustered according to whether tPA was administered or not (Figure 3a). Using the 533 gene probes as input to Prediction Analysis of Microarrays (PAM), 27 gene probes were found to optimally predict treatment with tPA (Additional File 3). Ten-fold leave-one-out cross-validation showed that these probes were able to correctly classify 12 of 12 rats based on treatment with tPA (Figure 3b).

Functional analysis was performed using IPA. The 533 gene probes mapped to 388 annotated genes and generated 316 network eligible genes. The top molecular functional pathways relate to cell-cell signaling, cellular organization and cell death (Table 2). These pathways predominantly reflect activities of immune cells and their interaction with endothelium. Top regulated genes included MMP9 (-3.36 fold), SERPINB6 (-4.16 fold), IL1R2 (2.14 fold), TLR2 (-1.69 fold), ICAM2 (-1.33), and ANXA2 (-1.21 fold).

**Table 2:** List of top 5 networks, canonical pathways and molecular functional pathways, for gene differentially regulated by tPA in the reperfused-MCAO group and permanent-MCAO group (p ≤ 0.01, fold change ≥ |1.2|).

**Reperfused-MCAO**					**Permanent-MCAO**	
			
	**Network**	**IPA Score**			**Network**	**IPA Score**
			
**1**	Carbohydrate Metabolism, Lipid Metabolism, Small Molecule Biochemistry	43			Gene Expression, Cell Cycle, Lipid Metabolism	41
			
**2**	Organismal Survival, Connective Tissue Development and Function, Tissue Development	43			Cellular Assembly and Organization, Cellular Function and Maintenance, Cancer	34
			
**3**	Lipid Metabolism, Molecular Transport, Small Molecule Biochemistry	39			Genetic Disorder, Metabolic Disease, Neurological Disease	25
			
**4**	Inflammatory Response, Connective Tissue Disorders, Inflammatory Disease	34			Cellular Compromise, Drug Metabolism, Endocrine System Development and Function	25
			
**5**	Cellular Compromise, Cell Morphology, Endocrine System Development and Function	22			DNA Replication, Recombination, and Repair, Cancer, Cell Cycle	22
			
			
	**Canonical Pathway**	**P-value**			**Canonical Pathway**	**P-value**
			
**1**	Nicotinate and Nicotinamide Metabolism	3.27E-04			FcgRIIB Signaling in B Lymphocytes	1.01E-02
			
**2**	Hypoxia Signaling in the Cardiovascular System	8.48E-04			p38 MAPK Signaling	1.89E-02
			
**3**	Role of NFAT in Regulation of the Immune Response	4.77E-03			Glucocorticoid Receptor Signaling	2.33E-02
			
**4**	B Cell Receptor Signaling	1.72E-02			Estrogen Receptor Signaling	3.09E-02
			
**5**	TREM1 Signaling	1.97E-02			Macropinocytosis	3.86E-02
			
			
	**Molecular Functional Pathway**	**P-value**			**Molecular Functional Pathway**	**P-value**
			
**1**	Cell-To-Cell Signaling and Interaction	7.10E-04 - 4.50E-02			Gene Expression	1.65E-04 - 1.29E-02
			
**2**	Cellular Assembly and Organization	7.10E-04 - 3.47E-02			Cell-To-Cell Signaling and Interaction	6.52E-04 - 4.37E-02
			
**3**	Cell Cycle	1.40E-03 - 3.93E-02			Cellular Movement	8.51E-04 - 4.56E-02
			
**4**	Cellular Function and Maintenance	1.97E-03 - 3.33E-02			Carbohydrate Metabolism	1.61E-03 - 3.81E-02
			
**5**	Cell death/Survival	2.09E-03 - 2.85E-02			Cellular Assembly and Organization	2.03E-03 - 4.69E-02

Key: The majority of the pathways identified reflect activities of immune cells and their interaction with endothelium. (IPA, Ingenuity Pathway Analysis; MCAO, middle cerebral artery occlusion; tPA, tissue plasminogen activator)

#### tPA gene expression in rats with permanent-MCAO

**Figure 4:**
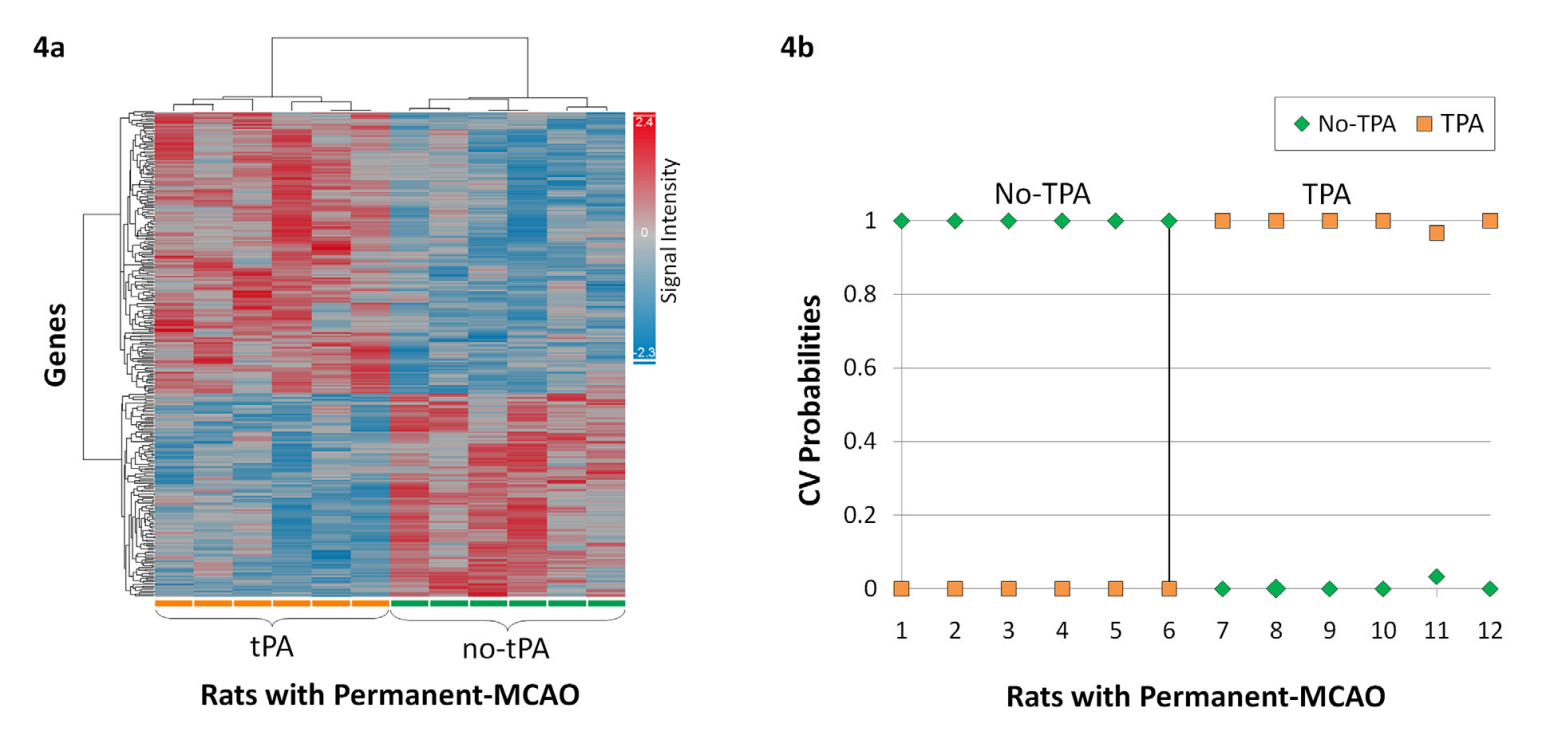
**Analysis of gene probes differentially regulated in the blood of rats with permanent-MCAO without reperfusion (p ≤ 0.01, fold change ≥ |1.2|)**. (4a) Hierarchical cluster plot showing gene probes clustering by treatment with tPA. (4b) Cross-validation with Prediction Analysis of Microarrays (PAM) of gene probes differentially regulated by tPA. The 62 gene probes correctly predict treatment with tPA. (Rats not treated with tPA are shown on the x-axis by numbers 1-6, rats treated with tPA are number 7-12; y-axis represents the cross-validated probability of the predicted class)

In rats with permanent-MCAO, treatment with tPA led to differential regulation of 293 gene probes at 24 hours (p ≤ 0.01, fold change ≥ |1.2|) (Figure 2) (Additional File 4). Of the 293 gene probes, 170 were up regulated (58.0%) and 123 were down regulated (42.0%). The 293 gene probes clustered by tPA administration (Figure 4a). Using the 293 gene probes as input to PAM, 62 probes were found to optimally predict treatment with tPA (Additional File 5). Ten-fold leave-one-out cross-validation showed that these gene probes were able to correctly classify 12 of 12 rats based on treatment with tPA (Figure 4b).

Functional analysis was performed using IPA. The 293 gene probes mapped to 181 annotated genes and generated 142 network eligible genes. The top molecular functional pathways were cell-to-cell signaling, cellular movement, and cellular organization (Table 2). These pathways predominantly reflect activities of immune cells and their interaction with endothelium. Top regulated genes include TIMP3 (+2.39 fold), ITGB2 (+1.21 fold), COL17A1 (+1.27 fold), GZMB (+1.74 fold), JAG1 (-2.09 fold), CSF-1 (-1.70 fold), SLP1 (-1.70 fold), FCGR2B (-1.51 fold), and PIK3CD (-1.31 fold).

## Discussion

tPA administration was associated with differential expression of 929 gene probes in the blood of rats with ischemic stroke. The genes expressed by tPA differed depending on whether or not ischemic stroke was accompanied by reperfusion. Most genes had functions related to modulation of immune cells. This data is important as it has potential implications for tPA treatment of ischemic stroke and understanding the pharmacogenomics of tPA.

### tPA and the Immune System

tPA modulated gene expression related to the immune system. This is not unexpected, given that immune cells are intimately involved in tPA related complications of BBB breakdown and HT of ischemic brain tissue [5]. Moreover, tPA has previously been reported to act on a number of immune cells, including mast cells and neutrophils.

Mast cells play various roles in ischemic stroke, including altering BBB permeability, hemorrhage formation, brain edema formation, and regulation of local neutrophil infiltration [14]. tPA promotes mast cell degranulation, thus enhancing release of vasoactive, proteolytic, and fibrinolytic substances. Mast cell stabilization with cromoglycate reduces tPA related brain hemorrhage, brain edema and neutrophil infiltration [15]. In our study, we identified a number of genes differentially regulated by tPA that affect mast cells, including those involved in mast cell activation (FCGR2B) and mast cell degranulation (COL17A, PIK3CD, ORAI1).

Neutrophils are recruited and activated early on following ischemic stroke. tPA has been shown to promote neutrophil degranulation [16,17]. Upon degranulation, neutrophils release preformed granules containing proteolytic enzymes, including matrix metalloproteinase-9 (MMP-9), that facilitate migration of leukocytes across the BBB to ischemic brain tissue [18]. The mechanisms by which tPA acts on neutrophils to mediate these effects are unknown. However, products of tPA clot degradation may contribute in part to neutrophil activation and infiltration [19]. A number of the identified tPA regulated genes in our study are associated with neutrophils. In the reperfused-MCAO group, tPA was associated with genes involved in neutrophil activation (SELL, ANXA1, SLPI) and neutrophil migration (MMP9, FGR, FCGR2B). In the permanent-MCAO group, genes involved in neutrophil attachment to endothelium (ITGB2) and genes involved in neutrophil recruitment (SLP1, CSF-1) was observed. Further study is required to delineate the effects of tPA on immune response in ischemic stroke, including determination of the specific immune cells and pathways affected by tPA. These potentially identify new targets to reduce tPA related complications in ischemic stroke and perhaps extend the therapeutic window of tPA.

### tPA and Immune-Endothelial Effects

Endothelial cells form an integral part of the BBB along with the basal lamina and astrocytic foot processes. tPA can affect endothelial cells in both a direct and indirect manner. Indirectly tPA modulates immune cells that interact with endothelium. tPA increases endothelial adhesion molecule expression including ICAM2, P-Selectin, and E-Selectin [20,21]. Direct effects of tPA on endothelial cells is mediated via the Lipoprotein Receptor Protein-1 (LRP-1). Binding of tPA to LRP-1 initiates a signal cascade that up regulates endothelial MMP-9 and disrupts the BBB [22-24].

Our study identified a number of differentially regulated genes involved in immune-endothelial signaling pathways, including genes involved in inflammatory cell adhesion, migration, and recruitment. In the reperfused-MCAO group, genes involved in immune-endothelial interaction include ICAM2, CEACAM1, ID1, and ACTN1. Notably, genes involved in immune-endothelial signaling were not as significantly affected by tPA in the permanent-MCAO group. This suggests that further study of genes involved in immune-endothelial interaction may provide insight into the increased risk of tPA related HT in subjects with reperfusion compared to permanent occlusion.

### tPA and MMP-9

MMP-9 has been studied in ischemic stroke at both the protein and RNA level in blood and brain. MMP-9 is a protease that degrades extracellular matrix and promotes BBB breakdown. Protein levels of MMP-9 increase in the blood early on after ischemic stroke onset, and this increase is enhanced by tPA [25,26]. In rats treated with tPA, the rise in blood MMP-9 peaks by 6 hours and return to baseline by 24 hours [25]. In human stroke patients treated with tPA, a similar rise blood MMP-9 occurs by 8 hours and with a return to baseline by 25 hours [26]. MMP-9 mRNA expression in the blood following tPA treated ischemic stroke remains poorly studied. We found MMP-9 mRNA to be reduced at 24 hours. This is consistent with an early rise of MMP-9 levels in the blood by 6-8 hours, followed by a reduction by 24 hours. This is in contrast to the pattern of MMP-9 expression in the brain, where levels remain elevated at the 24-30 hour time point [27,28]. Further study is required to better delineate MMP-9 mRNA expression patterns in blood, including the relationship with vascular reperfusion, duration of cerebral ischemia, treatment with tPA, and correlation with expression patterns of MMP-9 in cerebral tissue.

### tPA and Cerebral Reperfusion

The genes regulated by tPA in the blood were different depending on the status of vascular reperfusion. Rats reperfused after two hours of cerebral ischemia had a different tPA gene expression profile than rats with permanent-MCAO. Reperfusion of ischemic vasculature and cerebral tissue leads to cerebral ischemia/reperfusion (IR) injury. This injury includes glutamate-mediated excitotoxicity, oxidative stress, inflammation, and necrotic or apoptotic cell death [29]. Our study suggests that tPA modulates IR injury beyond the effects mediated by reperfusion alone. This modulation includes alteration of immune cells and immune-endothelial interaction. I/R injury is a complex process and its impact on clinical outcome in ischemic stroke is dependent on numerous factors including intensity and duration of ischemic insult, time to reperfusion, and the microcirculation [30]. Further study of tPA's effects on reperfusion may aid in identifying targets to reduce the contribution of tPA to I/R injury.

### tPA and Ischemic stroke

Blood gene expression profiling is a promising approach for diagnosing and understanding the pathogenesis of ischemic stroke [8,9]. In this study of rat ischemic stroke we were able to identify a gene expression profile associated with rat ischemic stroke. Some of the ischemic stroke genes were also regulated by tPA, indicating an interaction between ischemic stroke and tPA. However, many genes associated with ischemic stroke are not affected by tPA including BCL6, F5, and LY96 which were previously found to be predictive of ischemic stroke in humans [8]. Of the 1355 genes previously identified in humans to be differentially regulated by ischemic stroke, only 79 overlapped with the 929 tPA genes we identified in this study of rats [8]. This suggests that although tPA affects the gene expression profile in whole blood and should be considered when evaluating gene expression profiles in acute ischemic stroke, it is possible to distinguish tPA effects from the effects of ischemic stroke on gene expression. A gene expression profile can also be used to identify exposure to drug treatment. We were able to correctly predict tPA use in 12 out 12 rats with 27 gene probes in the reperfused-MCAO group and 62 gene probes in the permanent-MCAO group.

A strength of our study is the measurement of all known genes in the blood to determine those altered by tPA in ischemic stroke. A limitation is the analysis of blood gene expression only. tPA is known to mediate effects on endothelium and brain, thus further study is required to correlate changes in the blood with changes in these tissues. Additionally, we were only able to investigate a single dosage of tPA at a single time point. The equivalent dose for non-fibrinolytic effects of tPA in rats compared to humans remains unknown, therefore results need to be interpreted with this in mind. Finally, anesthesia was used in the experimental model. Although accounted for in the ANOVA model, anesthesia may still have had some effect on gene expression.

## Conclusions

A gene expression profile for tPA was identified in our rat model of ischemic stroke. The changes of gene expression by tPA reflect modulation of the immune cells. The patterns of gene expression by tPA were dependent on the status of cerebrovascular reperfusion. The majority of genes modulated by tPA in blood are distinct from those modulated by ischemic stroke. This suggests that in the blood of humans it should be possible to monitor gene expression profiles that correspond both to ischemic stroke and to the pharmacological effects of tPA.

## Methods

### 1. Animals

Forty-two male Sprague-Dawley rats weighing 280-350 grams (Charles River Labs, USA) were used in this study with a mean age of 2.4 months. The University of California at Davis Animal Care Committee approved the animal protocol in accordance with NIH guidelines. Animals were randomly assigned to one of seven experimental groups (n = 6 per group, N = 42 total): (1) Naïve; (2) Anesthesia; (3) tPA at 3 hours with no MCAO; (4) Reperfused-MCAO (2 hours of MCAO followed by 22 hours of reperfusion); (5) Reperfused-MCAO + tPA at 3 hours (6) Permanent-MCAO (24 hours MCAO with no reperfusion); and (7) Permanent-MCAO + tPA at 3 hours. Neurologic examinations were performed at 4 and 24 hours. The neurologic findings were scored on a four-point scale: 0, no neurologic deficit; 1, right Horner's syndrome; 2, failure to extend left forepaw fully; 3, circling to the left [31].

### 2. Focal ischemia

MCAO was produced using the intraluminal suture technique [31]. Rats were anesthetized with 3% isoflurane and maintained on 1.5% isoflurane in 100% oxygen. The right common carotid artery was exposed, and a 3-0 monofilament nylon suture was inserted into the external carotid artery and advanced into the internal carotid artery 20-23 mm beyond the carotid bifurcation until resistance was felt. MCAO was maintained for 2 hours in the reperfused-MCAO group and 24 hours in the permanent-MCAO group. Reperfusion was achieved by withdrawing the suture at 2 hours after MCAO onset. A heating blanket was used to maintain rectal temperature at 36.5°C to 37.5°C. Three hours following ischemia onset, tPA (3.3 mg/kg IP) was administered to rats in the tPA treatment groups. This dose of tPA is within the range of previous studies on rats of 3-10 mg/kg [32,33]. We opted for a dose of tPA on the lower end of this range to be more comparable to the dose used in rabbits and humans in the study of tPA's non-fibrinolytic effects. Recombinant human tPA used was a gift from Genentech Inc. (South San Francisco, CA). Animals were anesthetized with 3% isoflurane at 24 hours, and a blood sample drawn.

### 3. Sample processing and microarray hybridization

Blood was collected into PAXgene vacutainer tubes via cardiac puncture (PreAnalytiX, Germany). RNA was isolated using the PAXgene blood RNA kit (PreAnalytiX, Germany). Reverse transcription, amplification, and sample labeling were carried out using Nugen's Ovation Whole Blood reagents (Nugen Technologies, San Carlos, CA). Each RNA sample was hybridized and scanned according to the manufacturer's protocol onto Affymetrix Rat Genome 230 2.0 GeneChips (Affymetrix Santa Clara, CA). The arrays were washed and processed on a Fluidics Station 450 and scanned on a Genechip Scanner 3000. Microarray data have been deposited in the NCBI Gene Expression Omnibus (GEO) database Accession Number GSE21136.

### 4. Probe-level data analysis

Raw expression values (probe level data) were imported into Partek software (Partek Inc., St. Louis, MO) and processed using GC-RMA normalization [34]. Statistical analysis, principal components analysis, and hierarchical unsupervised clustering analysis were performed with Partek Genomics Suite 6.04. Probe lists were analyzed using PAM (Prediction Analyses of Microarrays) nearest shrunken centroids to identify the minimum number of gene probes able to differentiate specified groups [35]. PAM analysis uses a 10-fold leave-one-out cross-validation, meaning the model is fit to 90% of the samples and the class of the remaining 10% of samples are predicted. This procedure is repeated 10 times to compute the overall error in the model. Ingenuity Pathway Analysis (IPA, Ingenuity Systems^®^, http://www.ingenuity.com) was used to determine whether the numbers of genes regulated within given pathways or cell functions were greater than expected by chance.

### 5. Statistical analyses

To evaluate gene expression differences related to tPA, two analyses were performed. The first analysis was designed to identify gene expression associated with tPA administration and determine the relationship between tPA and ischemic stroke on gene expression. Using analysis of variance (ANOVA) the effects of tPA, ischemic stroke, anesthesia, and the interaction of tPA*ischemic stroke were evaluated. Unsupervised hierarchical clustering and principal components analysis were used to evaluate relationships between tPA treated and non-tPA treated rats. Gene probes with a p value ≤ 0.05 and a fold change ≥ |1.2| were considered significant.

The second analysis was designed to examine the genes regulated by tPA in rats as a function of reperfusion status. ANOVA was performed comparing tPA treated rats to non-tPA treated rats in the reperfused-MCAO groups. Similarly, ANOVA was performed comparing tPA treated rats to non-tPA treated rats in the permanent-MCAO groups. Given the smaller sample size relative to analysis 1, gene probes with a p value ≤ 0.01 and a fold change ≥ |1.2| were considered significant. Student's *t *tests were performed to determine differences between tPA treated and non-treated groups. The Mann-Whitney rank sum test was performed for analysis of neurological score. All data are presented as mean ± SE.

## Competing interests

The authors declare that they have no competing interests.

## Authors' contributions

The project was conceived by GCJ, BPA, XZ and supervised by FRS and PAL. All data analysis was carried out by GCJ with advice and input from BPA, BS, HX, RJT, DL and XZ. Animal surgeries were conducted by XZ and BPA. The RNA extraction was done by BPA and YT. The microarray gene expression experiments were done by RD. GCJ wrote the manuscript with input from all authors. All authors read and approved the final manuscript.

## Supplementary Material

Additional file 1 **List of genes regulated by tPA in rat ischemic stroke**. The 929 gene probes differentially regulated by tPA in rats with ischemic stroke (p ≤ 0.05, fold change ≥ |1.2|. Click here for file

Additional file 2 **List of genes regulated by tPA in reperfused-MCAO**. The 533 genes probes differentially regulated by tPA at 24 hours in rats with reperfused-MCAO, (p ≤ 0.01, fold change ≥ |1.2|). Click here for file

Additional file 3 **List of genes predictive of tPA treatment in reperfused-MCAO**. The 27 gene probes identified to optimally predict treatment with tPA in rats with reperfused-MCAO. The 27 probes were derived from the 533 gene probes in Additional File 2 using Prediction Analysis of Microarrays (PAM). Click here for file

Additional file 4 **List of genes regulated by tPA in permanent-MCAO**. The 293 gene probes differentially regulated by tPA at 24 hours in rats with permanent-MCAO (p ≤ 0.01, fold change ≥ |1.2|) Click here for file

Additional file 5 **List of genes predictive of tPA treatment in permanent-MCAO**. The 62 gene probes identified to optimally predict treatment with tPA in rats with permanent-MCAO. The 62 probes were derived from the 293 gene probes in Additional File 4 using Prediction Analysis of Microarrays (PAM). Click here for file
